# Photocatalytic Response of Flash-Lamp-Annealed Titanium Oxide Films Produced by Oblique-Angle Deposition

**DOI:** 10.3390/nano15090662

**Published:** 2025-04-26

**Authors:** Raúl Gago, Slawomir Prucnal, Francisco Javier Palomares, Leopoldo Álvarez-Fraga, Ana Castellanos-Aliaga, David G. Calatayud

**Affiliations:** 1 Instituto de Ciencia de Materiales de Madrid, Consejo Superior de Investigaciones Científicas, E-28049 Madrid, Spain; fjp@icmm.csic.es (F.J.P.); leo.alvarez@icmm.csic.es (L.Á.-F.); 2 Institute of Ion Beam Physics and Materials Research, Helmholtz-Zentrum Dresden-Rossendorf, D-01328 Dresden, Germany; s.prucnal@hzdr.de; 3 Departmento de Química Inorgánica, Universidad Autónoma de Madrid, E-28049 Madrid, Spain; ana.castellanos@icv.csic.es (A.C.-A.); david.gcalatayud@uam.es (D.G.C.); 4 Instituto de Cerámica y Vidrio, Consejo Superior de Investigaciones Científicas, E-28049 Madrid, Spain

**Keywords:** titanium oxide, oblique-angle deposition, flash-lamp-annealing, photocatalysis

## Abstract

We report the photocatalytic (PC) response of titanium oxide (TiO*_x_*) films grown by reactive DC magnetron sputtering under oblique-angle-deposition (OAD) and subjected to post-deposition flash-lamp-annealing (FLA). Under ballistic growth conditions, OAD yields TiO*_x_* films with either compact or inclined columnar structure as the deposition incidence angle (*α*) with respect to the substrate normal varies from zero to grazing. On the one hand, films produced for *α* ≤ 45° display a compact and opaque structure comprising the formation of nanocrystalline cubic titanium monoxide (*c*-TiO) phase. On the other hand, films grown at larger *α* (≥60°) display tilted columns with amorphous structure, yielding highly porous films and an increased transparency for *α* > 75°. For TiO*_x_* films grown at large *α*, FLA induces phase transformation to nanocrystalline anatase from the amorphous state. In contrast to *as-grown* samples, FLA samples display PC activity as assessed by bleaching of methyl orange dye. The best PC performance is attained for an intermediate situation (*α* = 60–75°) between compact and columnar structures. The obtained photoactivity is discussed in terms of the different microstructures obtained by OAD and posterior phase formation upon FLA.

## 1. Introduction

Titania or titanium dioxide (TiO_2_) is a relevant prototype photocatalyst [1,2]. In this way, the photocatalytic (PC) response of TiO_2_-based materials has been exploited in many fields ranging from pollutants decomposition [3], CO_2_ reduction [4], water treatment [5], water splitting [6], and many others. The broad range of applications partially rely on the tuneable electrical and optical properties of TiO_2_, which depend on intrinsic (structural phase and/or native defects) or extrinsic (doping) factors [7]. In particular, TiO_2_ materials can be found in different polymorphs, being anatase and rutile the most common phases. Generally, anatase displays a higher PC efficiency than rutile. First, anatase TiO_2_ has a larger bandgap than rutile, providing a slightly higher redox capability [8]. In addition, the indirect character of the anatase bandgap results in longer lifetime of the photogenerated carriers [9]. Additional features of nanocrystalline anatase that explain its higher PC activity compared to rutile rely on a much higher surface area (presence of more active sites) and higher concentration of oxygen vacancies (with enhanced charge separation efficiency) [8]. Under this framework, control over phase formation (comprising single and mixed phase materials) is a critical issue to tailor the final optoelectronic properties.

Another relevant factor for the PC performance is the effective surface (active) area. For that reason, TiO_2_ nanostructures has been widely studied [10]. Regarding samples in thin-film form, one remarkable way to obtain nanostructured coatings is by means of oblique angle deposition (OAD). Under such configuration, ballistic growth (comprising high directionality) and reduced surface diffusion (low temperature growth) at the substrate can be used to obtain porous films with separated and tilted nanocolumns [11]. The development of such morphology is the result of atomic shadowing processes that start with the formation of the initial agglomerates at the early growth stage and keeps operating during the further film growth evolution. The diameter of the nanocolumns is typically of the order of tens of nm, whilst their length is easily defined by the deposition time [12]. OAD has been applied to the production of nanostructured TiO_2_ films but, generally, the easiest approach relies on the growth of Ti films followed by an oxidation process to produce well-shaped oxide microstructures [13]. OAD TiO_2_ samples display remarkable PC activity and cyclability and, in general, their performance seems to be significantly higher than in the flat counterparts [14,15,16] and comparable to that of nanoparticle films [17]. The latter result highlights the role of roughness and porosity to produce TiO_2_ samples with high specific surface area [18,19]. In this case, critical aspects controlling the photoactivity of TiO_2_ OAD samples rely on the column spacing and length [16,20]. Under this approach, the overall PC performance of OAD TiO_2_ films has been tailored by adjusting the resulting morphology under varying incidence angles with or without simultaneous or sequential substrate movements [14,15,21].

The annealing of initially amorphous TiO_2_ has been studied as a cost-effective method for crystal growth and phase control, which has a strong impact on the PC efficiency. Thermal annealing has been applied to promote the PC of OAD TiO_2_ films [13] although special attention has to be taken to avoid eventual changes in the *as-grown* morphologies through relaxation processes. Ultrafast annealing approaches can help to further reduce the energy input in comparison to conventional annealing methods and promote crystal growth without damaging the microstructure. In this context, flash-lamp-annealing (FLA) is a non-isothermal and non-contact process where a rapid increase and quenching of the temperature occurs [22]. As already shown in monolithic TiO_2_ films [23,24], millisecond-range FLA from initially amorphous films offers a novel pathway for crystal growth and phase selectivity. The ultrafast thermal budget during FLA impacts on the transformation process of the metastable anatase phase into the thermodynamically more stable rutile phase, which is driven by controlling the solubility of the phase mixture [25]. In this way, FLA has been proven as a promising approach to fabricate highly photoactive TiO_2_-based materials [26].

In this work, we study the impact of FLA on the structure of sputtered TiO*_x_* films grown under OAD. The aim is to improve the structural order of the *as-grown* films by FLA and evaluate the PC response of the obtained microstructures. In this case, the presence of tilted columns and high porosity (roughness) are relevant aspects that could contribute to improve the PC response. The results evidence that FLA activates the PC response of the films and that the photoactivity promotion is a complex trade-off of several factors that come into play. In particular, the best PC performance is related to the formation of incipient nanocolumns together with interfacial issues due to presence of finely grained anatase phase together with subsurface reduced Ti^4+^ environments.

## 2. Materials and Methods

### 2.1. Sample Preparation

Titanium oxide (TiO*_x_*) films were grown by DC reactive magnetron sputtering from a high-purity (99.99%) 3” Ti target. The OAD experiments were carried out with deposition angles (*α*) from 0 to 85° in order to tune the film structure. The manipulator of the sample holder allows to set *α* with an accuracy of ± 5°. The deposition was performed simultaneously on Si(100) and sapphire substrates, and the Ti cathode was located at a distance of ~15 cm from the grounded substrates. The base pressure of the deposition chamber was 10^−4^ Pa and a mixture of Ar (99.9995% pure) and O_2_ (99.9995% pure) was used at a relatively low working pressure of 0.1 Pa to maximize the directionality of the deposition flux. The Ar/O_2_ ratio was set to 9/1 in order to obtain a relatively high deposition rate (i.e., working below the transition region of the reactive sputtering process with the target state closer to the metallic mode) to compensate the low effective deposition flux at large *α*. The plasma was generated by a DC signal with a power of 150 W. The growth was carried out for different times (increasing with *α*) to reach a similar thickness in the range of 150–200 nm for all the samples. The sample holder was water-cooled, keeping the substrates at room temperature during deposition.

The *as-grown* samples on Si substrates were subsequently processed by FLA for 23 ms at a continuous flow of O_2_ (99.999% purity) with an effective energy density of 40 J/cm^2^. Under this condition, the surface reaches a maximum peak temperature around 1000 °C, as extracted from simulations performed with the COMSOL software v6.1 [27]. The heating and cooling rates during millisecond FLA were in the range of 100 K/ms and 200 K/s, respectively. Further details about the FLA system can be found elsewhere [20,28].

### 2.2. Sample Characterization

The microstructure of TiO*_x_* films grown by OAD before and after FLA has been imaged by scanning electron microscopy (SEM) with a FEI Verios 460 instrument operating at 2 kV. Plan-view as well as cross-sectional images were acquired to evaluate the surface morphology and the growth evolution, respectively. The film thickness was extracted from the cross-sectional images. Additional (quantitative) information about the surface morphology was obtained by atomic force microscopy (AFM). The AFM images were acquired in tapping mode with a NanoObserver^®^ microscope from CSInstruments (Les Ulis, France).

Rutherford backscattering spectrometry (RBS) was used to determine the composition and atomic incorporation rate in *as-grown* samples. The measurements were carried out at *Centro de Micro-Análisis de Materiales* (CMAM) of the *Universidad Autónoma de Madrid* (Spain). The 5 MV Cockroft-Walton tandetron accelerator at CMAM was used to generate a 1.8 MeV He^+^ probing beam impinging under normal incidence with respect to the target. The energetic backscattered particles were detected with a silicon detector located at a scattering angle of 170° and the spectra were acquired for a total ion dose of 15 µC. The experiments were performed in “*random geometry*” to avoid channelling effects in the substrate signal. For quantitative analysis, the experimental RBS spectra were simulated with the SIMNRA software v7.00 [29].

Micro-Raman spectra were recorded at room temperature in backscattering geometry. Samples were excited with a 532 nm laser at a power of 10 mW and beam diameter of about 1 mm. The Raman signal was collected with a liquid He cooled Si-CCD in the 50–900 cm^−1^ range with a spectral resolution of 0.1 cm^−1^. Phase formation was examined by grazing-incidence X-ray diffraction (GI-XRD) using a D5000 diffractometer from BRUKER AXS (Billerica, MA, USA) with Cu-Kα radiation (wavelength of 1.5418 Å). The data were collected at an incidence angle of 0.5°. Finally, chemical analysis was extracted from X-ray photoelectron spectroscopy (XPS) in a Phoibos 150 electron spectrometer from SPECS (Berlin, Germany) using a non-monochromatic Al-Kα X-ray source. The energy calibration was carried out by setting the *C 1s* binding energy from adventitious carbon at 285.0 eV.

### 2.3. Wettability and Photocatalytic Properties

The wettability properties before and after FLA was studied with ultra-pure (Milli-Q) water using static contact angle measurements with a CAM 101 equipment from KSV Instrument Ltd. (Espoo, Finland). After stabilization of the droplets deposited on the surface, the static contact angle was measured. The extracted values were averaged over at least four measurements on each sample at different surface locations.

The PC assays were performed using a high-pressure Hg vapour lamp for UV–visible irradiation (250 W, HPL-N Philips, Amsterdam, The Netherlands) with an incident photon flux of 3.13 μE/cm^2^s. The PC activity was extracted from the photodegradation of aqueous solutions of methyl orange (MO) with a concentration of 10^−5^ mol/L. The samples were immersed in 10 mL of the aqueous solution using a glass beaker and then exposed to the light source. Aliquots from the suspension were taken periodically after different irradiation times and the concentration (degradation) of MO was determined by monitoring the change (decrease) in absorbance at 460 nm with a spectrophotometer Analytik-Jena Specord 200 Plus (Jena, Germany) equipped with both deuterium and tungsten lamps. Two side-effects were considered to avoid data misinterpretation: (*i*) the self-degradation of MO under irradiation and/or (*ii*) its adsorption on the surface of the samples. In order to disregard both scenarios, the solution stability was first verified by illuminating a blank solution (without photocatalyst) under the same experimental conditions and, secondly, by keeping the solutions with the photocatalysts under dark ambient. In both cases, no change in the MO concentration was detected. The tests were repeated three times for each sample, observing in all cases a good cyclability within a statistical uncertainty below 1%.

## 3. Results and Discussion

### 3.1. Microstructure of As-Grown OAD TiO_x_ Films

**Figure 1:**
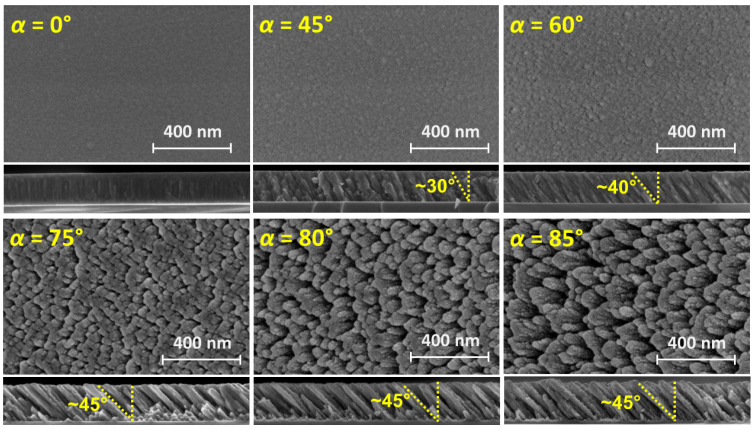
FE-SEM plan-view and cross-section images of TiO*_x_* films grown by OAD under different incidence angles, *α*. The formation of inclined columns is produced for *α* ≥ 60°. The column inclination (*β*) with respect to the surface normal is indicated in the cross-sectional images.

Figure 1 shows the plan-view and cross-section SEM images of *as-grown* TiO*_x_* films prepared under different *α* values. The images display a transition from compact films to a microstructure of tilted columns with the apex pointing toward the deposition source as *α* increases. The columnar growth is clearly defined for *α* ≥ 60°. For grazing incidence condition (*α* ≥ 75°), the morphology drives to the formation of more defined and isolated columns, leaving empty pores between them, as a result of an enhanced shadowing effect during growth under such geometry. The presence of pores at the surface can also be hinted from the darker zones in the plan-view SEM images. The observed microstructures are akin to that reported in OAD TiO*_x_* films produced by electron beam evaporation [30].

**Figure 2:**
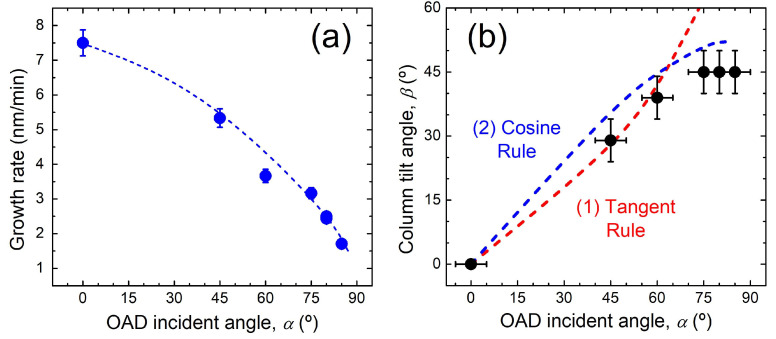
(**a**) Growth rate and (**b**) column inclination (*β*) of TiO_2_ OAD films as a function of *α*.

The film thickness extracted from the cross-sectional SEM images can be used to calculate the growth rate as a function of *α*. As shown in Figure 2a, the growth rate decreases with *α* as a result of the reduction in the effective flux of incoming particles towards the substrate (larger effective area). At grazing incidence, the relative reduction in the growth rate is remarkable (~80%). In addition, Figure 2b shows the tilting of the columns (*β*) as a function of *α.* The trend indicates a higher tilting with *α* followed by a saturation at *β* ~45° for grazing incidences (*α* ≥ 75°). An equivalent behaviour has also been found for other oxides such as zinc oxide [31]. The variation in the tilting angle has been studied with empirical analytical models where the “tangent” and “cosine” rules seen to apply for low and high α values, respectively [32]. This scenario clearly operates in the data displayed in Figure 2b. It should be noted that the prediction from these empirical models can be improved with material-dependent parameters [33] or, as shown for the case of evaporated TiO_2_, through numerical simulations considering the angular broadening of the deposition flux [34].

**Figure 3:**
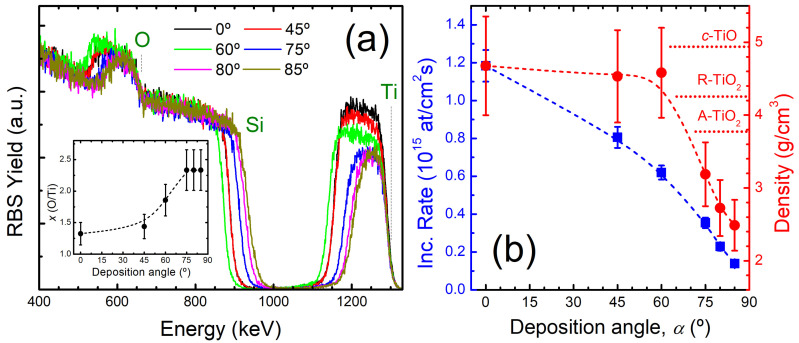
(**a**) RBS spectra of *as-grown* TiO*_x_* films produced by OAD under different *α* angles. The inset shows the composition (*x*) obtained from the simulation results. (**b**) Atomic incorporation growth rate (■) and density (●) of the films as a function of *α*.

Figure 3a shows the RBS spectra obtained from *as-grown* TiO*_x_* films. The signals from the film containing elements have been labelled in the figure for data interpretation, where heavier elements appear at higher energy. The Ti yield is proportional to the Ti content in the films. In this way, the Ti (O) content in the films progressively decreases (increases) with *α* until reaching a saturation regime for *α* ≥ 75°. The quantification of the O/Ti ratio (*x*), as obtained from the simulation of the RBS spectra, is displayed as inset in Figure 3a. Clearly, films produced for *α* ≤ 60° present *x* < 2 whereas, for *α* ≥ 75°, *x* is greater than 2. In the latter case, the values are above the TiO_2_ stoichiometry, which should be related to the relatively high porosity of the films and O_2_ embedded into the film microstructure. It should be noted that the incorporation of gas molecules during growth at low temperature is expectable during reactive magnetron sputtering [35,36]. The area of the Ti signal provides information about the atomic areal density (at/cm^2^) of the films, which decreases with *α*. The atomic areal density divided by the deposition time can be used to calculate the atomic incorporation rate as a function of *α*. The trend shown in Figure 3b indicates that the atomic deposition rate decreases with *α* following a behaviour that, obviously, matches that shown in Figure 2a for the growth rate. The films have similar thickness but the Ti band significantly shrinkages at *α* ≥ 75°. This trend indicates a change in the film density, as displayed in Figure 3b. It should be noted that the density of *c*-TiO is 4.95 g/cm^3^ whereas the density for the rutile and anatase polymorphs is 4.23 and 3.78 g/cm^3^, respectively [37]. The relatively high density (~4.5 g/cm^3^) for *α* ≤ 60° correlates with the formation of Ti-rich films, whereas porous films are clearly produced for *α* ≥ 75°. The density (porosity) decreases (increases) with *α*, yielding a maximum decrease of ~55% for *α* = 85°. This strong reduction evidences the formation of a large portion of voids at high *α* and, obviously, this trend correlates with the formation of more spaced columns and less compact films observed by SEM. In the case of tilted nanocolumns, it is obvious that the density will be closely related to the column separation in the film and, ideally, equal to the column width divided by the column spacing [30]. As observed here, the latter assumption implies a sharp fall in the density at high *α*. Experimentally, an equivalent relative decrease in the density as in Figure 3b with *α* has been reported for SiO_2_ films produced by OAD [38], which implies a 0.4–0.5 volume fraction of voids for grazing incidence geometries. In the case of TiO_2_ films produced by OAD, Li et al. [13] reported for vertical columns (grown under simultaneous substrate rotation) void fractions in the same range; however, in that case, the film density decreased from 1.9 to 1.5 g/cm^3^ with *α*.

### 3.2. Effect of FLA in OAD TiO_x_ Films

**Figure 4:**
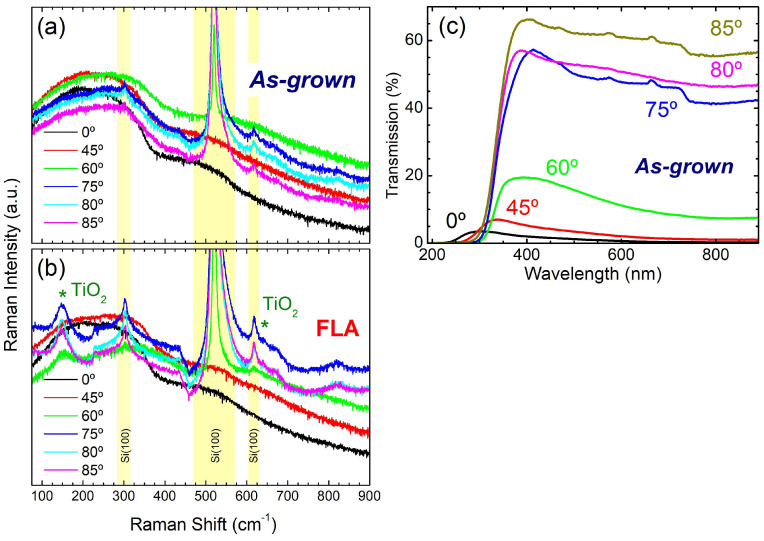
Raman spectra (**a**) before and (**b**) after FLA for TiO*_x_* films produced by OAD at different *α* values. The presence of vibration bands from anatase TiO_2_ (marked with *) in FLA samples are indicated. (**c**) Optical transmission of equivalent *as-grown* samples deposited on sapphire substrates.

Figure 4a,b compare the Raman spectra of *as-grown* and FLA samples, respectively. The *as-grown* samples display similar structure, with broad and featureless spectra that suggest the amorphous or disorder structure of the films. In *as-grown* films, none of the characteristic vibration bands from Ti-O phases can be observed. Also, samples grown with *α* ≤ 45° are rather opaque, as derived by the absence of bands related to the underlying Si substrate. On the contrary, these bands become evident for *α* ≥ 60° as a result of the higher film transparency. These assumptions have been confirmed by the optical transmittance in *as-grown* films on sapphire substrates, which is displayed in Figure 4c. On the other hand, Figure 4b shows that FLA induces the appearance of Raman bands associated with anatase TiO_2_ (marked with *) for *α* ≥ 60°. The most noticeable feature appears at 150 cm^−1^ together with a weak signal at 640 cm^−1^, both features being related to *E_g_* vibration modes [39]. Note that the band at 150 cm^−1^ is relatively weak at *α* = 60° but becomes more pronounced at higher *α*.

**Figure 5:**
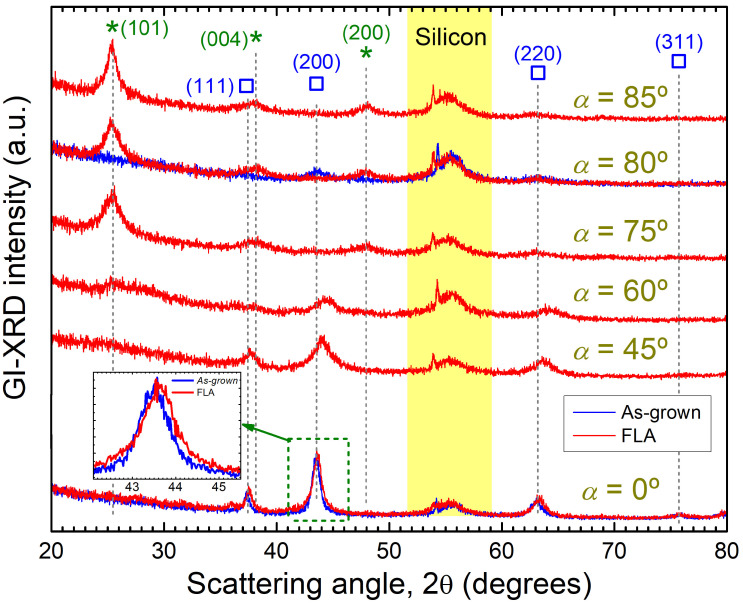
GI-XRD diffractographs of OAD TiO*_x_* films produced at different *α* values after FLA. The corresponding data from *as-grown* samples are also shown for *α* = 0° and 80°. Symbols * and □ are markers for the corresponding (*hkl*) peaks from anatase TiO_2_ and *c*-TiO phases, respectively.

Additional information about phase formation has been extracted from GI-XRD. Figure 5 displays the diffractographs obtained for TiO_x_ films after FLA. The GI-XRD pattern from as-grown samples at α = 0° and 80° are also shown for discussion. In the case of as-grown films, only reflections from the cubic titanium monoxide (c-TiO) phase can be identified. The signal from this phase is strong at near-normal incidence but its contribution seems to decrease progressively in films produced at larger α. Note that this phase is still present (but to a minor extent) even in the as-grown sample at grazing-incidence (α = 80°). In compact films, the c-TiO phase seems to be rather stable upon FLA since similar diffractographs are obtained in the sample produced at α = 0° after FLA and in the as-grown state. However, the corresponding peaks of c-TiO move to larger angles after FLA, suggesting a lattice contraction of the cubic structure as a result of the thermal modification (e.g., annihilation of defects). The contribution of c-TiO is still noticeable in the GI-XRD patterns of FLA samples produced at α ≤ 60°. In this case, the aforementioned lattice contraction of the cubic phase is more evident, together with a peak broadening indicating smaller cubic grains and/or higher stress as α is increased. For α ≥ 75°, FLA induces the transformation of the dominant amorphous structure into nanocrystalline anatase TiO_2_, in analogy to previous reports on compact TiO_2_ films [21,24]. Analysis of the peak widths through the Scherrer formula [40] indicates that the anatase grains produced by FLA in the OAD TiO_x_ films are around 10 nm. It is also relevant that for α ≥ 75°, no remnants of the as-grown c-TiO phase are detected after FLA (see the sample produced at α = 75°). In the film grown at α = 60°, GI-XRD shows a transition region where still a minor portion of c-TiO is present, together with the formation of fine-grained anatase phase.

**Figure 6:**
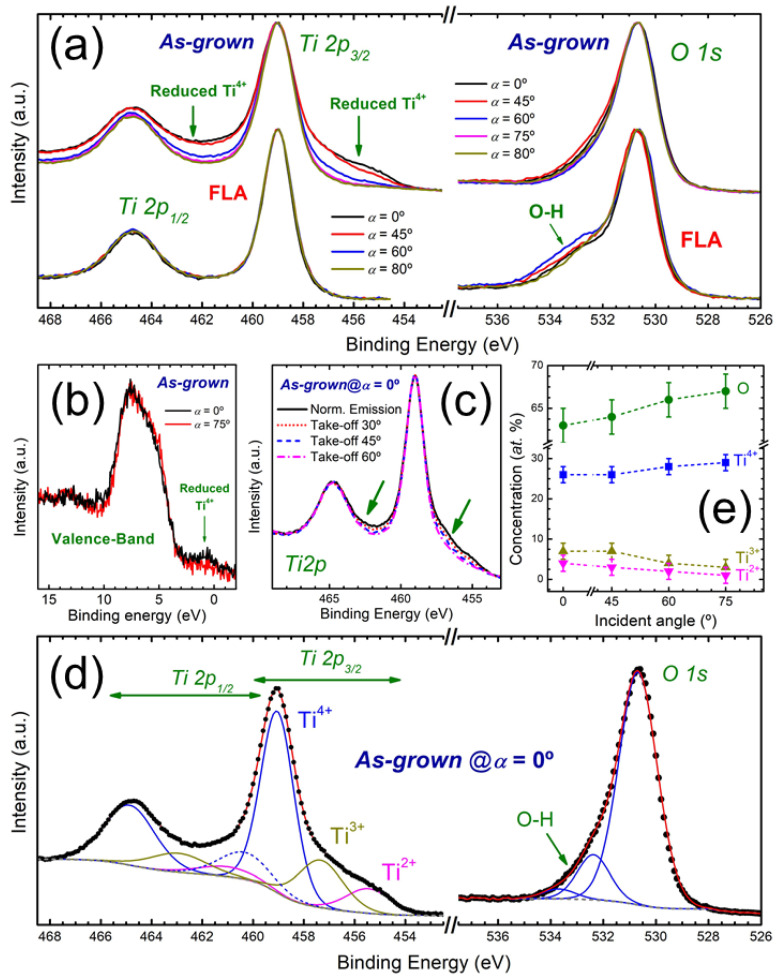
(**a**) XPS *Ti 2p* and *O 1s* core-level spectra of OAD TiO*_x_* films before (top) and after (bottom) FLA. (**b**) XPS valence band for *as-grown* films at *α* = 0° and 75°. (**c**) *Ti 2p* spectra for the *as-grown* film at *α* = 0° with different measurement *take-off* angles (i.e., sampling depths). (**d**) Fitting analysis of the XPS core-levels. (**e**) Atomic contributions in *as-grown* samples extracted from the fitting results.

The GI-XRD analysis indicates a trend in the Ti oxidation state from Ti^2+^ to Ti^4+^ in the films grown below and above *α* = 60°, respectively. In order to study this issue in more detail, the *as-grown* and FLA samples have been analyzed by XPS. The XPS *Ti 2p* and O *1s* core-level spectra from *as-grown* and FLA samples are shown in Figure 6a. Emission from reduced oxidation states should appear at lower binding energies with respect to Ti^4+^ (458.5 ± 0.1 eV) [41]. Hence, the presence of reduced states (Ti^2+^ and Ti^3+^) is readily evident at the *Ti 2p_3/2_* of *as-grown* samples from the filling of states below 457 eV. Accordingly, also note the corresponding signal increase at the low binding energy side of the *Ti 2p_1/2_* peak, filling the region between the *Ti 2p* doublet. On the contrary, the presence of reduced Ti^4+^ is absent in the case of FLA samples as an indication of TiO_2_ formation. Regarding the *O 1s* core-level, the signal is dominated by Ti–O bonds from O-lattice, whereas the high binding energy tail is attributed to the presence of oxygen in the form of hydroxides, carbon-oxygen species and water molecules adsorbed on the sample upon exposure to atmosphere [42]. This tail is more noticeable in FLA samples since they have been exposed to air for a longer period. The presence of reduced Ti^4+^ in *as-grown* samples can also be confirmed from the XPS valence band spectra displayed in Figure 6b where samples produced with *α* of 0° and 75° are compared. In this case, new intermediate states appear within the band-gap for *α* = 0°, of which, the emission is characteristic of reduced states [7].

Regarding the oxidation state of Ti atoms, the presence of Ti^2+^ in the *as-grown* films produced at low *α* (≤ 45°) is expectable from the detection of *c*-TiO by GI-XRD. However, the *Ti 2p_3/2_* spectra are dominated by the contribution of Ti^4+^, which can be attributed to surface oxidation. Under this scenario, intermediate Ti^3+^ states may also be expected. The TiO_2_ surface termination has also been verified by tilting the sample so that different electron emission angles and probing depths are analyzed. Hence, higher take-off angles (from normal emission) result in a higher contribution from the most external surface layers in the XPS signal. As shown in Figure 6c for the *as-grown* sample with *α* = 0°, the data confirm that the outermost layers are completely oxidized in the form of TiO_2_. As indicated above, annealed samples display a complete oxidation of the surface within the range of the XPS probing depth (at least, up to a few nm’s). It should be noted that oxygen vacancies tend to diffuse easily with a thermal activated barrier, where the more favourable pathway seems to be towards subsurface states [43]. Hence, considering the higher stability of TiO_2_, it is expectable the evolution of the samples towards a TiO_2_-terminated surface through ageing or thermal treatments. In any case, the GI-XRD results indicate that reduced Ti^4+^ sites are still present at larger depths in FLA samples produced for *α* ≤ 60°.

A quantitative contribution from the different oxidation states in *as-grown* samples can be extracted by fitting analysis of the XPS spectra. The components used for the fitting are shown in Figure 6d considering the illustrative case of the *as-grown* sample produced with *α* = 0°. The contribution from Ti^4+^, Ti^3+^ and Ti^2+^ is clearly supported by the fitting. The chemical shifts (position) and full width at half maximum (FWHM) of the components for each oxidation state are in agreement with previously reported data [39]. It should be noted that several constraints should apply for the different fitting parameters. First, the orbital splitting (~5.7 eV), ratio (2:1) and relative width between the *Ti 2p_3/2_* and *Ti 2p_1/2_* components have been fixed. Also, an additional satellite in the Ti^4+^ contribution to the *Ti 2p_3/2_* states has been considered (dashed line), which is normally attributed to inelastic scattering [44]. Regarding the XPS *O 1s* core-level, the spectra have been fitted with three components attributed to Ti–O bonds (main and more intense peak) together with adsorbed surface oxygen from hydroxyl groups, water molecules and carbon-oxygen species [45]. The quantitative results from the fitting are shown in Figure 6e. As anticipated by the spectral line shape, the contribution of reduced Ti^4+^ decreases with *α*, being almost negligible above *α* ≥ 75°. In parallel, the oxygen concentration increases as the structure moves towards stoichiometric TiO_2_.

**Figure 7:**
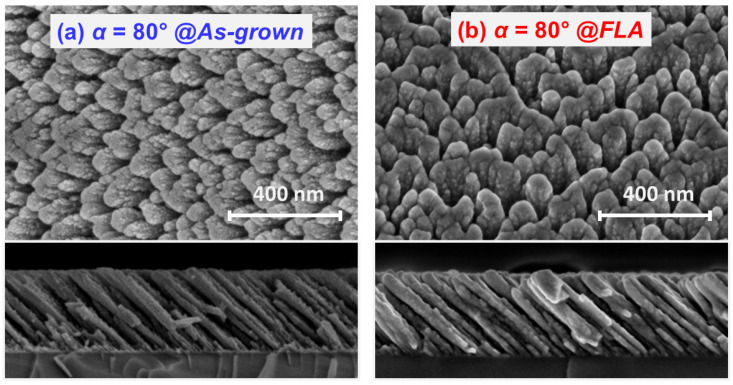
Comparison of the morphology of a TiO_x_ film grown with α = 80° (**a**) before and (**b**) after FLA as assessed by FE-SEM plan-view and cross-section images.

**Figure 8:**
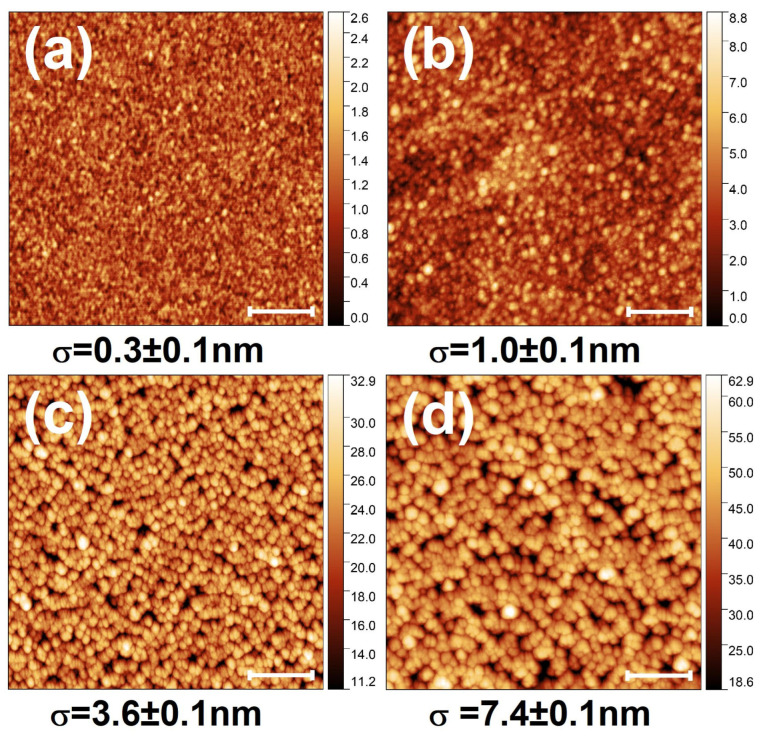
AFM 2 × 2 μm^2^ micrographs of OAD TiO*_x_* films after FLA produced with *α* of (**a**) 0°, (**b**) 60°, (**c**) 75° and (**d**) 80°. The rms roughness (σ) of each surface is also indicated. The scale bar corresponds to 500 nm.

In order to see the impact of FLA in the morphology of the OAD films, Figure 7a,b display the SEM images of the sample grown with *α* = 80° before and after FLA, respectively. It can be seen that the FLA treatment does not disrupt significantly the *as-grown* columnar features. After FLA, the inclined and isolated columns are still clearly evident as in the pristine case. The induced phase formation upon FLA can be hinted by the more rounded structures and a less defined texture, resulting in an overall smoother topography. There is also a slight broadening of the nanostructures as a result of phase formation. In particular, the column’s apex increases from the 30–40 nm range in the *as-grown* sample to 40–50 nm upon FLA. Additional (quantitative) information of the surface morphology after FLA has been attained through AFM. The images from FLA films produced with different *α* values are displayed in Figure 8a–d. In agreement with SEM, the films show a compact structure at low *α* (a–b) and the formation of a porous and rough surface under grazing configurations due to the tilted columnar growth (c–d). As indicated by the rms roughness (σ) values, the surface roughens with *α* comprising a significant increase in the presence of inclined columns. Note that the surface features and roughness become larger as *α* moves to a more grazing geometry due to the promotion of geometrical shadowing effects. In this case, a nearly two-fold roughness increase is obtained by changing *α* from 75° to 80°.

### 3.3. Photocatalytic Assesses

**Figure 9:**
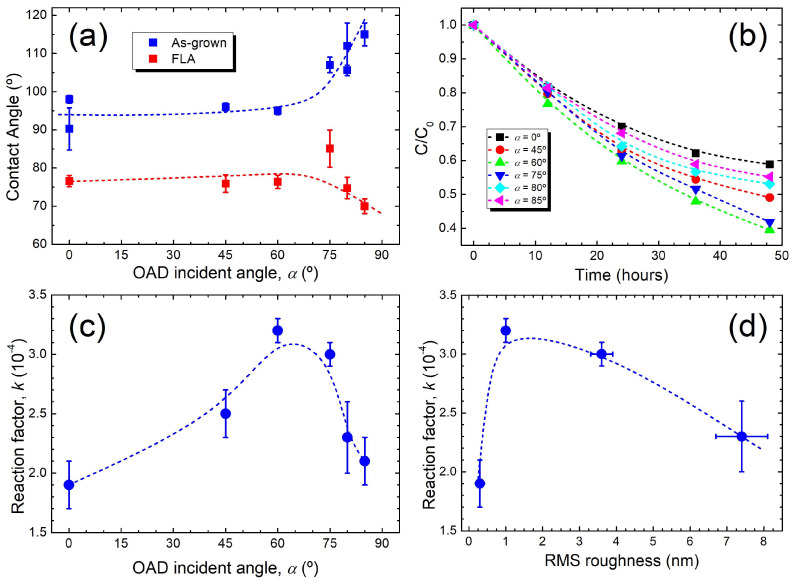
(**a**) Wettability of OAD TiO*_x_* films produced at different *α* values before and after FLA. (**b**) PC bleaching of MB for OAD TiO*_x_* samples after FLA. (**c**) Reaction rate factor (*k*) as a function of *α*. (**d**) Correlation between *k* and the surface roughness extracted from AFM.

Figure 9 illustrates the surface properties of the TiO*_x_* films. First, Figure 9a shows the change in wettability (surface energy) as a function of *α* before and after FLA. In *as-grown* samples, the contact angle remains constant at ~95° up to *α* = 60° and then increases sharply as a result of a well-defined columnar growth. The contact angle is significantly reduced to ~75° upon FLA, imprinting a clear hydrophilic character of the surface. This change may be relevant for the PC performance since *as-grown* samples show no PC activity. On the contrary, Figure 9b shows that FLA samples have a significant photoactivity as determined by MO bleaching. The corresponding reaction factor (*k*) has been extracted, assuming a first-order kinetic model described as C/C_0_ ~ exp (−*k*t) [46]. The variation in k as a function of *α* is shown as inset in Figure 9c. It is shown that the bleaching rate of MO increases with *α* until a maximum *k* is achieved around 60–75°, being 1.7 larger than for the *α* = 0° case. Then, the photoactivity drops for grazing-incidence angles, with *k* values slightly higher than in the compact film produced at *α* = 0°. A similar qualitative and semi-quantitative behaviour of the PC efficiency with *α* has been reported for TiO_2_ OAD films produced by electron beam evaporation and air-annealed [14]. In this case, the maximum efficiency was obtained at *α* = 70° for thin and close-packed nanocolumn arrays, with a 2.5-fold increase in *k* with respect to the sample produced at *α* = 0°. The efficiency then drops at larger *α* similarly as in Figure 9c but to a minor extent than in our case. The reduction in *k* at large *α* in Ref. [14] is attributed to the formation of thick and isolated columns, which would lead to a decrease in the surface area with respect to close-packed morphologies. The latter assumption has indeed been observed experimentally through porosity measurements in metal oxide films produced by OAD [47].

The present results demonstrate the use of non-contact FLA as an efficient method for the activation of the PC response of OAD TiO_2_ films. However, it is surprising that the maximum efficiency takes place at *α* = 60° where the film remains relatively dense (Figure 3b) and the tilted columnar growth only starts to be defined. Moreover, the anatase phase after FLA is not even detected by XRD and there are still traces of the *c*-TiO phase (see Figure 5). Here, surface roughness also does not seem to be the dominant factor since, as shown in Figure 9d, rougher samples do not display the highest *k*. XPS also reveals that the surface of all FLA samples is TiO_2_-terminated so the surface chemistry cannot explain the different behaviours. Though, the maximum efficiency does correlate with the transition region from Ti-rich films (with the formation of the *c*-TiO phase) to those with a dominant amorphous TiO_2_ character, the latter transforming into the nanocrystalline anatase phase upon FLA. Under this scenario, the optimum PC activity in our films could arise from the synergy between the columnar growth, incipient (anatase) grains and residual content of reduced TiO_2_ (at the subsurface region). In this context, it should be noted that highly responsive films can be obtained from finely grained nanocrystalline anatase [24]. In addition, reduced TiO_2_ can act as supplier of excess electrons from band-gap states that can affect the PC properties [48]. Note that such states have been detected in the valence-band spectra obtained from XPS (see Figure 6b). In any case, it should be noted that the OAD films after FLA with well-defined tilted nanocolumns and anatase grains still exhibit a considerable activity. The tilted columnar growth provides higher specific surface but the lateral grain area exposed (and the more photoactive) is defined by the column width (a few tens of nm’s). This may be a limiting factor since the final grain size attained after FLA plays crucial role in the PC response, as previously reported for the case of monolithic TiO_2_ films [24]. As already mentioned, other relevant characteristics affecting the PC of OAD films are the spacing between columns [16] and their length [20]. Hence, the production of alternative architectures modulating the columnar microstructure and thickness together with defect interface engineering (reduced TiO_2_) could be a potential strategy for further increase the PC response of such nanostructured films.

## 4. Conclusions

In conclusion, TiO*_x_* thin films with either compact or columnar microstructures have been produced by reactive DC magnetron sputtering under OAD as a function of *α*. For each microstructure, the samples are dominated by nanocrystalline *c*-TiO or amorphous TiO_2_ environments, respectively. Post-deposition FLA treatments induce the transformation to nanocrystalline anatase TiO_2_ from the amorphous state whereas the *c*-TiO phase in compact films is rather stable under the thermal treatment. XPS shows that the surface of all films is TiO_2_-terminated and that the surface oxidation process is further promoted after FLA. AFM also shows a pronounced increase in surface roughness with *α* that correlates with the emphasis of tilted nanocolumns. PC tests carried out by bleaching of MO dyes indicate that *as-grown* samples do not display a significant photoactivity, in contrast to the highly responsive FLA samples. The highest PC yield in FLA samples is attained for *α* in the range of 60–75°, that is, in the transition region between compact and nanocolumnar films. The surface roughness seems to play a minor role in the PC activity, which seems to be stronger where the nanocolumnar growth is incipient. In this case, the presence of finely grained anatase phase and reduced Ti^4+^ at subsurface regions seems to play a cooperative role.

## Data Availability

The raw data supporting the conclusions of this article will be made available by the authors on request.
